# AFAP1 Is a Novel Downstream Mediator of TGF-β1 for CCN2 Induction in Osteoblasts

**DOI:** 10.1371/journal.pone.0136712

**Published:** 2015-09-04

**Authors:** Youngjin Cho, Rachel Silverstein, Max T. Geisinger, Stephen Martinkovich, Holly Corkill, Jess M. Cunnick, Sonia L. Planey, John A. Arnott

**Affiliations:** The Commonwealth Medical College, Scranton, Pennslyvania, United States of America; Northwestern University, UNITED STATES

## Abstract

**Background:**

CCN2 acts as an anabolic growth factor to regulate osteoblast differentiation and function. CCN2 is induced by TGF-β1 and acts as a mediator of TGF-β1 induced matrix production in osteoblasts and Src is required for CCN2 induction by TGF-β1; however, the molecular mechanisms that control CCN2 induction in osteoblasts are poorly understood. AFAP1 binds activated forms of Src and can direct the activation of Src in certain cell types, however a role for AFAP1 downstream of TGF-β1 or in osteoblats is undefined. In this study, we investigated the role of AFAP1 for CCN2 induction by TGF-β1 in primary osteoblasts.

**Results:**

We demonstrated that AFAP1 expression in osteoblasts occurs in a biphasic pattern with maximal expression levels occurring during osteoblast proliferation (~day 3), reduced expression during matrix production/maturation (~day 14–21), an a further increase in expression during mineralization (~day 21). AFAP1 expression is induced by TGF-β1 treatment in osteoblasts during days 7, 14 and 21. In osteoblasts, AFAP1 binds to Src and is required for Src activation by TGF-β1 and CCN2 promoter activity and protein induction by TGF-β1 treatment was impaired using AFAP1 siRNA, indicating the requirement of AFAP1 for CCN2 induction by TGF-β1. We also demonstrated that TGF-β1 induction of extracellular matrix protein collagen XIIa occurs in an AFAP1 dependent fashion.

**Conclusions:**

This study demonstrates that AFAP1 is an essential downstream signaling component of TGF-β1 for Src activation, CCN2 induction and collagen XIIa in osteoblasts.

## Introduction

Connective tissue growth factor (CCN2; formerly CTGF) is a 38kDa, cysteine rich, extracellular matrix protein that is involved in osteogenesis [1, 2]. CCN2^-/-^ null mice exhibit numerous defects in the craniofacial, axial, and appendicular skeleton as the result of impaired bone formation/mineralization [3]. CCN2 is expressed in active osteoblasts, is produced and secreted by osteoblasts [4, 5] and acts to promote osteoblast proliferation, matrix production and differentiation [4, 6–11]. CCN2 levels are stimulated by the potent, multifunctional, osteogenic growth factor transforming growth factor-beta 1 in numerous cell types (TGF-β1) [5, 11, 12]. One of the major effects of TGF-β1 on osteoblasts is its ability to stimulate the production and secretion of ECM [13–16], however the mechanisms or downstream effector genes that mediate this response are not understood. In osteoblasts, we recently demonstrated that CCN2 is stimulated by TGF-β1 and that CCN2 mediates TGF-β1 induced ECM synthesis [5, 11, 12]. The signaling pathways that control TGFβ1 induction of CCN2 in osteoblasts have only begun to be characterized, and we have recently demonstrated the involvement of Src [17].

Src is the founding molecule of a family of non-receptor tyrosine kinases that, when activated, are involved in numerous physiological and pathological processes including cell proliferation, survival angiogenesis and matrix secretion [18–20]. Src can be activated downstream of the TGF-β1 receptor [21, 22] or indirectly activated through integrin-mediated cell attachment induced by TGF-β1 [23–26]. We have previously demonstrated that TGF-β1 activates Src in osteoblasts and that TGF-β1 induction of CCN2 requires Src [17]. We have further showed that Src is required for TGF-β1 induced Erk activation and Smad activation/nuclear translocation [17, 27]. Although it is clear that Src is required for TGF-β1 induction of CCN2, the exact mechanism by which TGF-β1 activates Src in osteoblasts is not clearly understood.

Actin filament-associated protein 1 (AFAP1, AFAP-110) is the prototypical member of a family of three structurally related proteins: AFAP1, AFAP1 like 1 (AFAP1L1), and AFAP1 like 2 (AFAP1L2, XB-130). AFAP1 was discovered over two decades ago as a binding partner for oncogenic Src [28]. AFAP1 is a substrate of cSrc as well as Protein Kinase C (PKC) and harbors a binding site for PKC family members [29] and SH2 and SH3 binding motifs for cSrc [30, 31]. AFAP1 regulates actin filament cross-linking [32], invadosome formation/stability [29, 33–35] and cell contractility [36]. Thus one proposed role for AFAP1 is that it acts as an adaptor protein that directs the localization of kinases that regulate actin cytoskeletal organization [32, 37]. AFAP1 is upregulated in certain cancers and AFAP1 expression is associated with higher grades of prostate cancer [38]. Using AFAP1^-/-^ null mice we were the first to demonstrate a novel physiological role for AFAP1 in lactation [39]. These studies demonstrated that AFAP1 is required for the spatial and temporal regulation of cSrc activity in the normal breast during lactation to establish copious milk production at parturition and, specifically, required for milk fat production [40]. Although we are beginning to understand a physiological role of AFAP1 and its role in directing cSrc activity in the normal breast, potential roles for AFAP1 in other tissues and cells with abundant expression of AFAP1 have yet to be characterized. Moreover, our knowledge on upstream (receptors) and downstream signaling components/target genes that are involved in AFAP1 signaling remains incomplete.

Considering that AFAP1 is an important regulator of Src activity and that Src activity plays a central role in relaying TGF-β1 signaling to induce CCN2 expression and controls osteoblast functions including ECM production, we hypothesized that AFAP1 plays a role in the TGF-β1 signaling pathway and the regulation of Src activity in osteoblasts. Thus, this study characterizes the role of AFAP1 in regulating Src activation and CCN2 induction downstream of the TGF-β1 receptor in osteoblasts.

## Results

### AFAP1 is expressed in differentiating osteoblasts and up-regulated by TGF-β1

**Fig 1:**
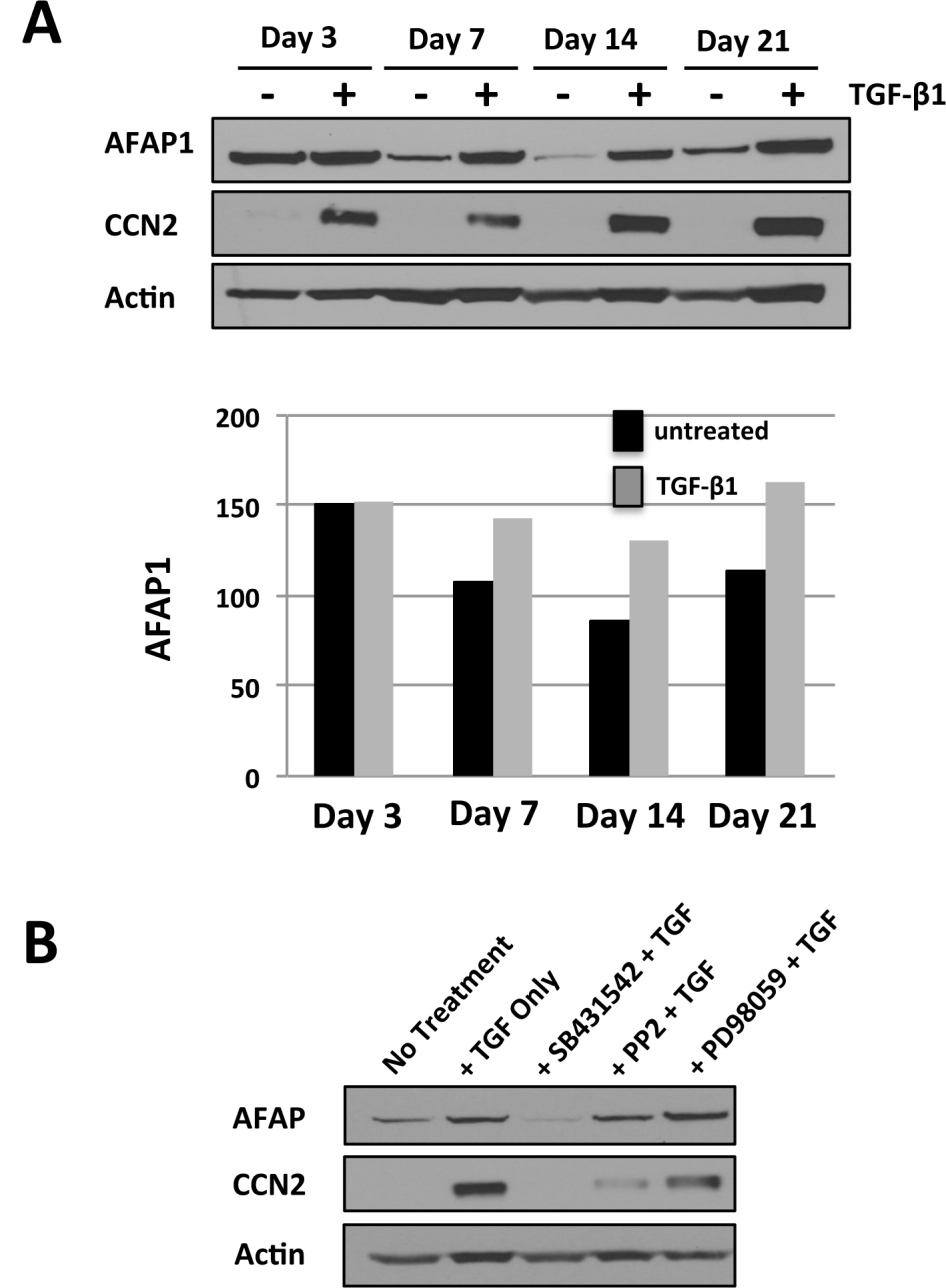
AFAP1 expression and induction by TGF-β1 in differentiating primary osteoblasts. (**A**)The data show that AFAP1 protein is expressed at all stages of osteoblast differentiation with the greatest found during osteoblast proliferation (day 3) and that AFAP1 protein is induced by TGF-β1 at day 7, 14 and 21. Primary osteoblasts were isolated and cultured under differentiating conditions for 3, 7, 14 or 21 days. Cells were either serum starved and either treated with TGF-β1 (5ng/ml) (+) or mock treated (TGF-β1 diluent) (-) for 24hrs. Western Blot was used to assess AFAP1, CCN2 and Actin protein levels. The data is representative of three independent experiments. (**B**) Osteoblasts were pre-treated with either SB431542 (10μM), PP2 (10μM), or PD98059 (10μM) for 24 hrs prior to TGF-β1 treatment for 24 hrs. We then measured AFAP1 and CCN2 expression by Western Blot. Western Blots are representative of triplicate determinations.

The three stages of osteoblast differentiation in primary osteoblast cultures have been well-characterized and include an initial period of cell proliferation until the cells reach confluency (~day 7), followed by a phase of matrix production and maturation (~day 7–14), and ending with a stage of mineralization in which mineral deposition accrues in the matrix (~day 14–21). We sought to assess the temporal pattern of AFAP1 expression in differentiating primary osteoblast culture and to determine if TGF-β1 was capable of inducing AFAP1 expression at different time points within the spectrum of osteoblast differentiation. Western Blot analysis revealed that AFAP1 protein expression occurred in a biphasic pattern with maximal expression levels occurring during osteoblast proliferation (~day 3), reduced expression during matrix production and maturation (~day 14–21), an a further increase in expression during mineralization (~day 21) (Fig 1A). This expression pattern suggests that AFAP1 plays a role early during differentiation for osteoblast proliferation. When we treated the cells with TGF-β1, AFAP1 protein expression was induced at day 7, day 14 and day 21 stages, but levels at day 3 were similar with or without treatment (Fig 1A., lanes marked by “+”). To confirm that AFAP1 expression was indeed the result of TGF-β1 treatment, we used day 7 osteoblasts as this was the earliest time point where AFAP1 induction by TGF-β1 was seen. Osteoblasts were pre-treated with 1) SB431542, the potent inhibitor of TGF-β1 receptor (TβRI), 2) PP2, a Src kinase inhibitor with moderate TβRI inhibition [41], or 3) PD98059, a MEK/ERK inhibitor that has no known inhibitory effects on TGF-β1 receptors. We then measured AFAP1 and CCN2 expression by Western Blot and found that SB431542, the potent TβRI inhibitor, completely suppressed TGF-β1 induced-AFAP1 protein expression. PP2 had a more moderate effect and PD98059 had no effect on AFAP1 protein expression by TGF β1 (Fig 1B). All of the inhibitors reduced CCN2 expression by TGF β1 stimulation as we previously reported [42], since each block a critical component required for TGF-β1 induced CCN2. These results demonstrated a temporal expression for the AFAP1 protein in osteoblast culture and also showed that TGF-β1 is a relevant upstream signaling molecule that controls AFAP1 expression in osteoblasts.

### AFAP1 is required for TGF-β1 induced Src activation in osteoblast

**Fig 2:**
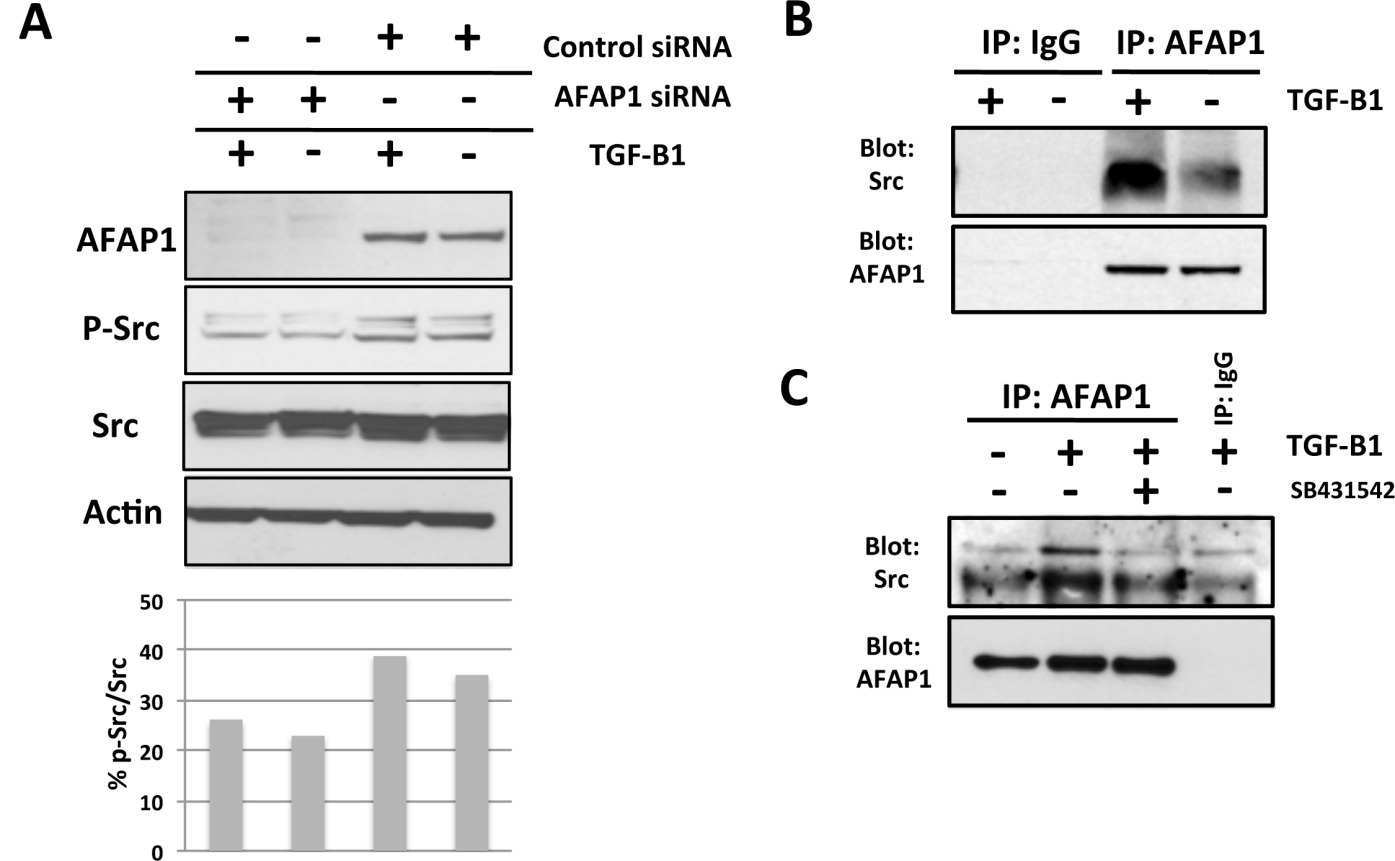
AFAP1 signaling is necessary for TGF-β1 induced Src activation in osteoblast. The data show that AFAP1 is required for Src activation by TGF-β1 (A) and that AFAP1 binds Src in osteoblasts in a TGF-β1 inducible manner (B). Methods: (A) Primary osteoblasts were pretreated with AFAP1 siRNA (siRNA) or non-targeting control siRNA (control) and the treated with TGF-β1 (5ng/ml; 2hrs) (+) or mock treated (-) and AFAP1, pTyr416-Src (pSrc) and Actin were assessed by Western blot. (B) Primary osteoblasts were treated with TGF-β1 (5ng/ml; 24hrs) (+) or mock treated (-) and IP with AFAP1 or IgG and blotted with Src or AFAP1 The data show that AFAP1 is required for Src activation by TGF-β1 and that AFAP1 binds Src in osteoblasts in a TGF-β1 inducible manner.

We previously showed that in osteoblasts TGF-β1 activates Src and TGF-β1 induction of CCN2 requires Src [17]. Since AFAP1 binds to Src in response to a specific upstream stimulus and is required for directing Src activity in the breast [39], we wanted to determine whether AFAP1 binds to Src in response to TGF-β1 and whether it is required for Src activation downstream of TGF-β1 in osteoblasts. When we treated osteoblast cultures with TGF-β1 we found that Src was activated (phosphorylated on tyrosine 416 in the activation loop of the kinase domain) in a TGF-β1 dependent manner (Fig 2A). Inhibition of AFAP1 expression using AFAP1 specific siRNA impaired Src activation by TGF-β1 (Fig 2A) and suggested that AFAP1 directs the activation of Src in osteoblasts. In order to determine if Src and AFAP1 form a complex as a result of TGF-β1 treatment, we treated osteoblasts with TGF-β1 or PBS (vehicle control), immunoprecipitated AFAP1 with an AFAP1 specific antibody and then probed for Src and AFAP1 by Western blotting. We found that TGF-β1 treatment increased complex formation between Src and AFAP1 (Fig 2B) compared to the vehicle control treatment. To confirm that this complex formation was a downstream of TGF-β1 receptor, osteoblasts were pretreated with SB431542 and the experiment was repeated as above. We found that SB431542 treatment impaired complex formation between Src and AFAP1 (Fig 2C). These results demonstrate that AFAP1 responds to TGF-β1 by forming a complex with Src in osteoblasts resulting in the activation of Src.

### AFAP1 is required for CCN2 induction by TGF-β1 in osteoblasts

**Fig 3:**
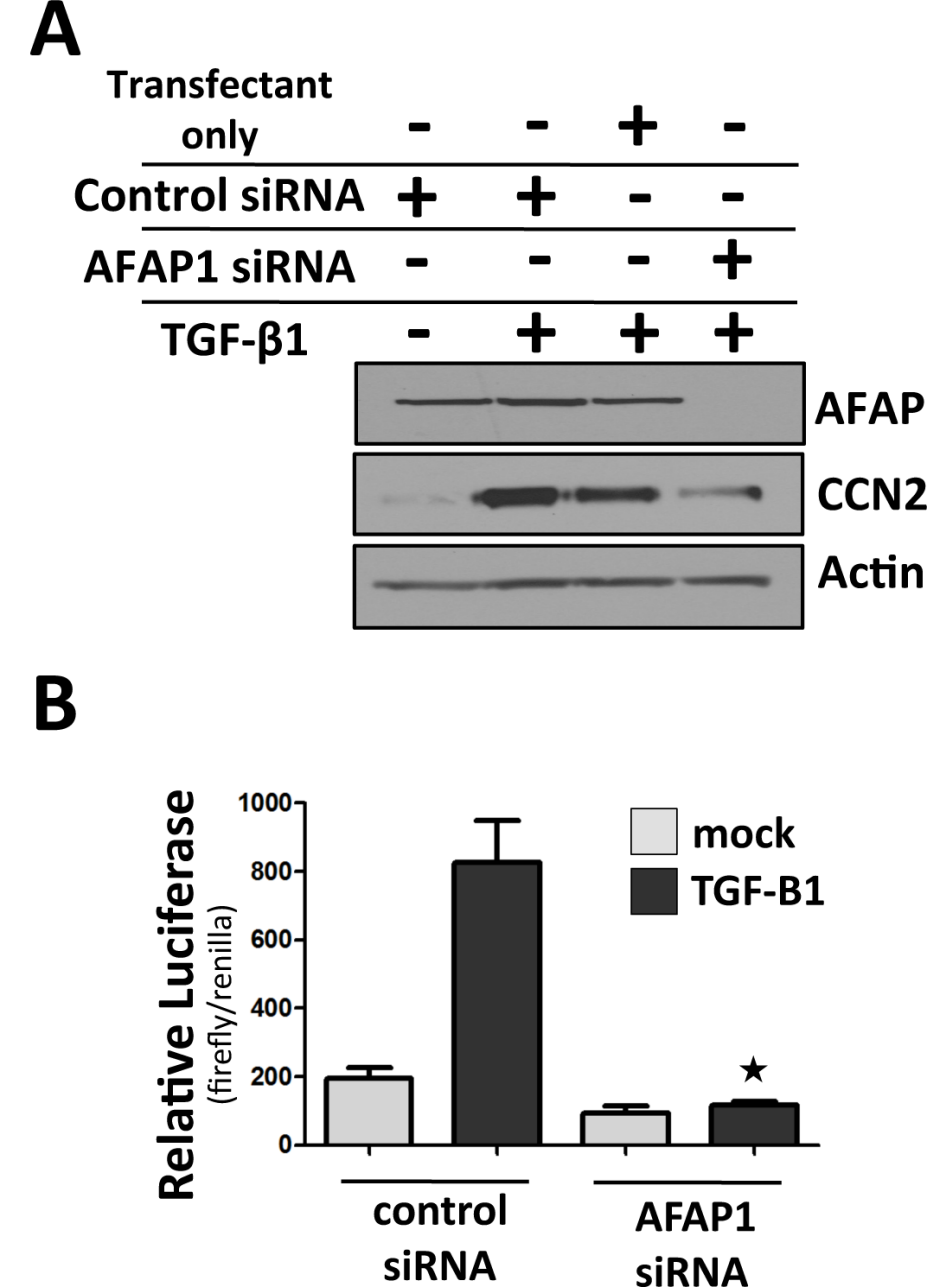
AFAP1 is required for CCN2 induction by TGF-β1. The data show that blocking AFAP1 expression with siRNA impairs CCN2 protein and promoter induction by TGF-β1 in primary osteoblasts. Additionally, CCN2 induction by TGF-β1 is impaired in osteoblasts derived from AFAP1^-/-^ mice. Methods: **(A)** Primary osteoblasts were pretreated with AFAP1 siRNA (siRNA) or non-targeting control siRNA (control) or transfection reagent only (transfectant only) and then treated with TGF-β1 (5ng/ml; 24hrs) (+) or mock treated (-) and AFAP1, CCN2 and Actin were assessed by Western blot. (**B**) Primary Osteoblasts were treated as in A, co-transfected with a full length CCN2 proximal promoter reporter, serum starved and then treated with TGF-β1 (5ng/ml) for 24hrs. Luciferase is expressed as a ratio of firefly/renilla. (SEM, n = 6) star symbol = p<0.05 compared to TGF-B1 +, control siRNA sample.

We have previously demonstrated that blocking Src expression/activity impairs CCN2 promoter activation and protein expression in response to TGF-β1 treatment in osteoblasts [27, 42]. To determine if AFAP1 plays a role in CCN2 induction by TGF-β1, we inhibited AFAP1 expression in osteoblasts (for these experiments 3 day osteoblast cultures were used) using AFAP1 specific siRNA and then treated the cells with TGF-β1. When AFAP1 expression was blocked with AFAP1 siRNA, TGF-β1-induced CCN2 protein expression was significantly reduced in osteoblasts, as determined by Western blotting (Fig 3A). We then pre-treated osteoblasts with AFAP1 specific siRNA, co-transfected osteoblasts with a CCN2 promoter reporter, treated the cells with TGF-β1, and measured CCN2 promoter activation by the luciferase assay. We found that TGF-β1 induced CCN2 promoter activation was abolished in AFAP1 siRNA pretreated osteoblasts, compared to that in osteoblasts treated with non-targeting siRNA control (Fig 3B). Taken together, these results demonstrate that AFAP1 is a relevant regulator of CCN2 expression downstream of the TGF-β1 receptor in osteoblasts.

### AFAP1 is required for TGF-β1-induced bone matrix protein production in osteoblasts

**Fig 4:**
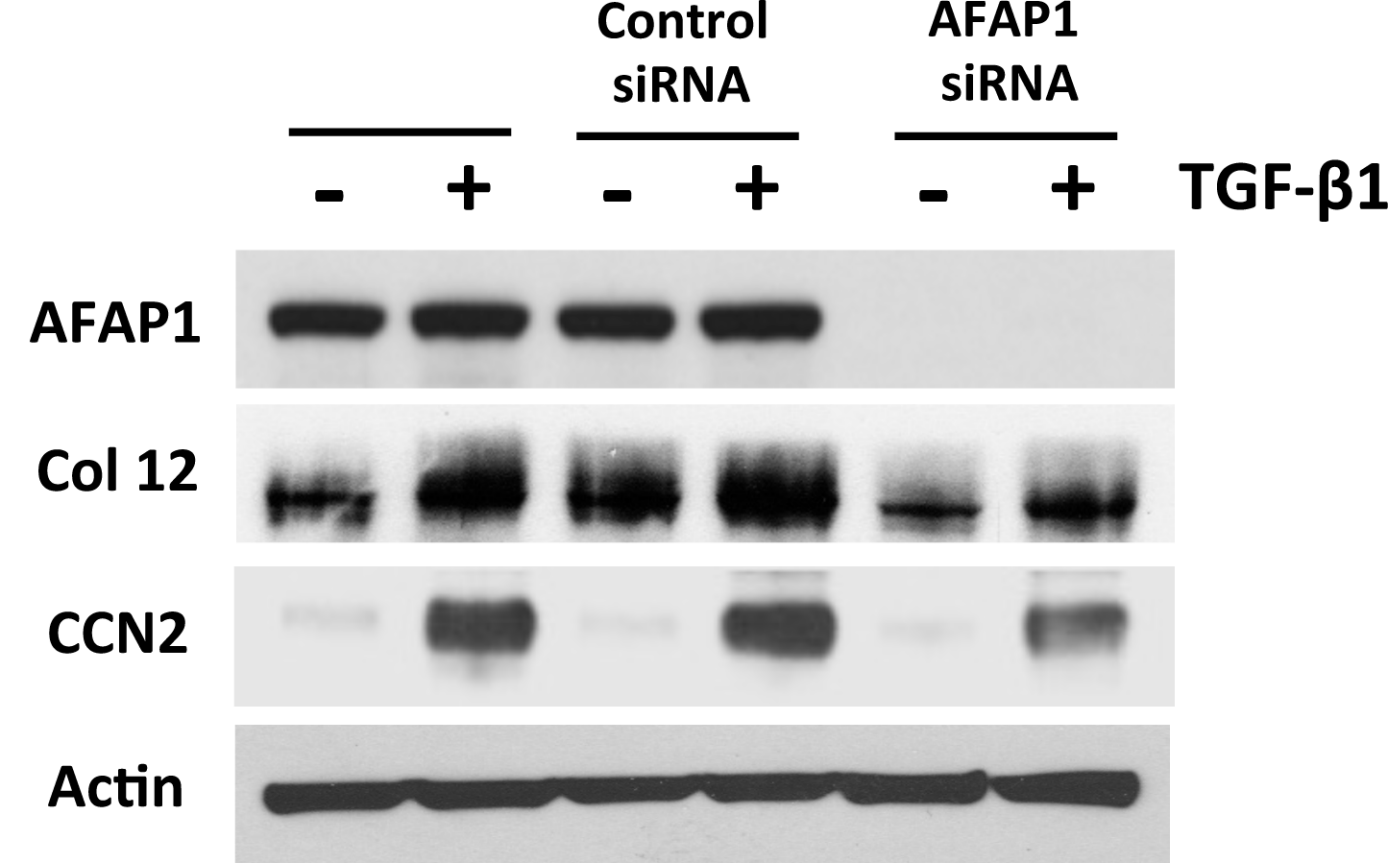
AFAP1 is necessary for TGF-β1 induced Col XIIa. The data show that blocking AFAP1 expression with siRNA impairs Col XIIa protein expression. Primary osteoblasts were pretreated with AFAP1 siRNA (siRNA) and then treated with TGF-β1 (5ng/ml; 24hrs) (+) or mock treated (-) and Col XIIa, AFAP1, CCN2 and Actin were assessed by Western Blot. Western Blots are representative of triplicate determinations.

We have previously demonstrated that CCN2 is an essential downstream mediator for the TGF-β1-induced, extracellular matrix (ECM) protein collagen type I in osteoblasts [43]. Additionally, using a yeast two-hybrid screen, we found that Collagen XIIa as one of several candidate molecules that interacted with AFAP1 with high probability (unpublished data). Considering 1) our above result showing that AFAP1 is important for CCN2 induction by TGF-β1, 2) that CCN2 mediates TGF-β1 induced ECM expression, 3) and preliminary data to support the connection between AFAP1 and the ECM protein, collagen XIIa, we tested if blocking AFAP1 using siRNA resulted in decreased production of ECM proteins such as collagen I, osteonectin, and collagen XIIa. These results demonstrate that the expression of both collagen XIIa was induced by TGF-β1 treatment in osteoblasts. However, the pretreatment of osteoblast with AFAP1 siRNA significantly suppressed this induction of collagen XIIa by TGF-β1 (Fig 4). The expression of osteonectin and collagen I was not affected by AFAP1 siRNA (unpublished result). These results indicated that AFAP1 is required for TGF-β1 induced ECM protein expression, particularly that of specific collagen proteins.

## Discussion

AFAP1 is considered as an adaptor protein that directs the activity and the location of cSrc and as an effector protein that crosslinks actin filaments. The function of AFAP1 has been studied mainly in prostate cancer and breast cancer where AFAP1 contributes to the progression of cancer by regulating the adhesion of cancer cells [38, 44]. We were the first to report the normal physiological function of AFAP1 to regulate Src activity in during lactation [39]. However, the role of AFAP1 in normal physiology or in development in other tissues still requires further investigation. In particular, the upstream input signals that regulate the function of AFAP1 and the downstream signaling pathways transduced by AFAP1 remain elusive. In this study, we present the novel finding that AFAP1 is relevant to osteoblast function by contributing to the TGF-β1 signaling pathway, a prominent anabolic pathway in bone physiology. We demonstrate that TGF-β1 is an upstream input signal that increases AFAP1 expression and induces the complex formation between AFAP1 and cSrc. Furthermore, we have identified the downstream pathways and target genes regulated by AFAP1 in osteoblasts. We demonstrated that AFAP1 is required for cSrc activation upon TGF-β1 stimulation and that AFAP1 is required for CCN2, collagen XII production upon TGF-β1 stimulation. This is the first study to uncover the role of AFAP1 in osteoblast physiology. Given numerous physiological and pathological roles of TGF-β1 and CCN2 signaling, this study also provides broad implications for the role of AFAP1 in other potential physiological/pathological conditions such as bone homeostasis and fibrosis/wound-healing.

CCN2 is an important factor in the induction and control of osteogenesis. CCN2 is highly expressed in active osteoblasts during osteogenesis [1], during fracture healing [2, 3] and during bone formation and regeneration [4]. Additionally, recombinant forms of CCN2 can elicit osteoinductive responses in bone, enhancing osteoblast differentiation, and inducing bone matrix protein deposition [2, 5]. TGF-β1 is a potent inducer of CCN2 in various connective tissue cell type including osteoblast. Upon induction, CCN2 mediates the various downstream effects of TGF-β1 on cell proliferation, migration, adhesion, and matrix production in specific cell types. The specific functions of the signaling axis between TGF-β1 and CCN2 also differ depending on the cell types [45–51]. The osteoblast-specific signaling mechanisms that control CCN2 expression has recently began to be clarified. We have previously showed that TGF-β1 activates Src kinase in osteoblasts and Src activity is required for CCN2 induction by TGF-β1 in osteoblasts [27, 42]. Src functions as a TGF-β1 signal transducer in different cell types [22, 23, 26, 52, 53] and Src activation following TGF-β1 treatment can occur as either a direct result of TGF-β treatment [21, 22] or indirectly through integrin-mediated cell attachment induced by TGF-β1 [23–26]. In this study, we identified AFAP1 as another molecular requirement for TGF-β1 signaling in osteoblast, necessary for Src activation and for CCN2 induction. These studies further support the role of Src as a key kinase downstream of TGF-β1 signaling in osteoblasts.

We found that reducing the expression of AFAP1 using AFAP1 siRNA impaired Src activation after TGF-β1 treatment in osteoblasts. This finding is consistent with other previous reports concerning the involvement of AFAP1 in binding to activated forms of Src [39, 54]. In the inactive state, Src kinases assume an auto-inhibitory conformation mediated by intramolecular interactions of the SH2 and SH3 domains and a tyrosine phosphorylation site on the carboxy-terminus of the protein and the activation of Src requires engagement with the appropriate substrates [55]. AFAP1 contains motifs that enable the interaction with the SH2 and SH3 domains of Src. AFAP1 normally exists in an auto-inhibited conformation and when activated AFAP1 interacts with Src in an SH3-dependent fashion [32–34, 54]. AFAP1 functions in a combinatorial fashion with other partners for its interaction with Src and for subsequent downstream signaling and biological activity. We have previously shown that, in response to phorbol ester, AFAP interacts with PKCα to activate cSrc, leading to the formation of actin-rich structures such as podosomes [34, 56]. We also showed that Protein Kinase C (PKC) isoforms (PKCα, PKCβ1, PKCγ and PKCλ) were each able to bind to the amino terminal pleckstrin homology (PH1) domain of AFAP1 [29, 32]. PKCα will phosphorylate AFAP1 upon PKCα binding[29] and PKCα will affect conformational changes of AFAP1 that correlate with an ability to activate Src in an AFAP1-dependent manner [32, 34, 54].

Interestingly, PKC isoforms have been previously shown to be involved in TGF-β1 induction of CCN2 in mesangial cells [57], hepatocytes [58] and lung fibroblasts [59] and in animal models CCN2 induction was associated PKCβ2 activation [60]. However, one report found that mRNA levels of CCN2 were inhibited by the activation of PKC, but stimulated by the inhibition of PKC and tyrosine kinase [61]. Future studies will focus on the role of AFAP1 downstream of TGF-β1 and other partners necessary for Src activation with special focus on the role of PKC for this signaling in osteoblasts.

TGF-β1 is known to influence bone metabolism through effects in both osteoblasts and osteoclasts. Specifically, in osteoblasts, TGF-β1 recruits and induces the proliferation of osteoblast precursors and inhibits their apoptosis and can modulate expression of factors that control the formation and activation of osteoclasts [62]. In general, TGF-β1 signals through a generic Smad mediated pathway involving Smads 2, 3 and 4 [63], although the signaling pathways that mediate TGF-β1 induction of CTGF can vary depending on the cell type being examined [64]. Ligand induced activation of the TGF-β1 receptor results in the binding of Smads 2 and 3 to the receptor and subsequent Smad phosphorylation, resulting in release from the receptor and complex formation with Smad 4 [65]. TGF-β receptors can also activate Smad-independent signaling pathways that can regulate Smad activation and function [66]. MAPKs (Erk1/2, p38 and Jnk) represent another group of downstream signaling transducers of TGF-β1 that have been shown to directly regulate CTGF expression in some cell types [57, 67–72]. We previously showed that Smads 3 and 4 and Erk are required for CTGF induction by TGF-β1 in osteoblasts [42]. In addition, we also previously showed that Src is required for TGF-β1 induced Erk and Smad activation as well as Smad nuclear translocation in osteoblasts [17, 27]. Interestingly, AFAP1 contains two Pleckstrin Homology (PH) domains; one amino terminal (PH1) and one carboxy terminal (PH2). Both PH domains potentially direct the association of AFAP1 to WD-40 (a.k.a. beta-transducin) repeat–containing proteins, which are known to associate with the TGF-β1 receptor and facilitate activation of Smads and other downstream effecter proteins [66]. However, blocking AFAP1 with AFAP1 siRNA did not affect Erk or Smad 3 activation in our hands (unpublished data). As an adaptor of Src, AFAP1 is expected to direct specific activity of Src in a temporally and spatially controlled manner. Therefore, while AFAP1 is clearly required for Src activation and CCN2 induction upon TGF-β1 treatment, it is possible that the pool of Src that requires AFAP1 may not contribute to Smad and Erk activation. Rather, the AFAP1 and Src interaction may be required for yet unknown downstream signaling pathways to induce CCN2 expression in osteoblast. It is also possible that AFAP1 may function along a collateral pathway that is Src-independent. Further studies are necessary to determine the full compliment of molecules involved in the TGF-β1-AFAP1-CCN2 pathway.

A major function of TGF-β1 during osteoblast differentiation is to stimulate production of the extracellular matrix components that compose osteoid [14, 73]. In this study, treatment of osteoblasts with TGF-β1 significantly enhanced the expression of type XII collagen and this induction of extracellular matrix proteins was effectively inhibited by treating cells with AFAP1 siRNA. Combined with the results that AFAP1 siRNA blocked CCN2 production upon TGF-β1, these results are consistent with our previous studies where blocking CCN2 expression impaired TGF-β1 induced ECM protein synthesis [11]. These results further support the role of AFAP1 as a required downstream mediator of TGF-β1 for ECM protein induction in osteoblasts. ECM protein production in osteoblasts is a complex process where array of kinases and transcription factors come into play. For example, it is known that PKC, a known binding partner of AFAP1, also can regulate ECM protein production such as fibronectin and osteocalcin in osteoblast and chondrocytes respectively [74, 75]. Therefore, while the diminished CCN2 expression as the result of blocking AFAP1 may be the likely explanation for the reduced ECM protein synthesis, we cannot rule out the possibility that AFAP1 affects ECM protein production independent of CCN2. We are currently further delineating the exact mechanism through which AFAP1 affect collagen production.

In conclusion, this study demonstrates that a normal physiological role of AFAP1 is to mediate the downstream signaling pathway of TGF-β1 to induce CCN2 induction and extracellular matrix protein production, the prominent anabolic pathway in osteoblast. This study further supports the role of AFAP1 as an adaptor for Src to direct the activity of the kinase upon receiving a specific input signal, TGF- β1.

## Materials and Methods

### Ethics Statement

No human subjects were used in this study. This study was approved by the IACUC board of The Commonwealth Medical College, Scranton PA. (IACUC approval #13–07). All animals were handled according to national and international guidelines following the principles in the NIH Guide for the Care and Use of Laboratory Animals (U.S. Department of Health and Human Services, Publ. No. 86–23, 1985) and in accordance with principles established in the Weatherall report.

### Reagents

Transforming Growth Factor-β1 (TGF-β1) was purchased from Calbiochem (EMD Millipore, Billerica, MA) and reconstituted as 1μg/ml in 4mM HCl with 0.1% bovine serum albumin. Anti-actin antibody was purchased from Sigma (Sigma Aldrich, St. Luis, MO). Anti-CTGF, anti-collagen I and anti-collagen XII antibodies were purchased from Santa Cruz (Santa Cruz, CA). Anti-Src antibodies (clone GD11) were from BD Bioscience and antibodies specific for active form of Src (clone pY416) were from cell signaling Technologies (Boston, MA). CCN2 promoter reporter was constructed as previously described [17]. The generation and use of Anti-AFAP1 polyclonal antibodies, used in immunoprecipitation, was as described [28]. Mouse monoclonal anti-AFAP1 antibodies used in western blotting were purchased from BD Bioscience (San Jose, CA). Inhibitors SB431542, PP2, PD98059 were purchased from EMD Millipore (Billerica, MA).

#### AFAP1 siRNA

Osteoblasts were transfected with 100 pmol of siRNA specific for AFAP1 (pool of 4 siRNAs, GCGCCUUCCUGUUGCGUAA, UCACGUACAUCCCGAGAGA, CCAACAUCCUGCUUCGAAU, GAAAAGAGGCCCUGCGGAA, Dharmacon, Thermo Fisher Scientific, Pittsburgh, PA) or 100 pmol control Luciferase siRNA (CGUACGCGGAAUACUUCGAdTdT, Dharmacon) using Lipofectamine and Plus reagent (Invitrogen, Life Technologies, Carlsbad, CA) according to the manufacturer’s instructions.

#### AFAP1-GFP

The generation GFP AFAP1 fusion protein construct was previously reported [37]. GFP expression vector (Clonetech, Mountain View, CA) or GFP AFAP1 was transfected to osteoblast using Dharmafect (Dharmacon, Thermo Fisher, Pittsburgh, PA).

### Source of Animals

Primary osteoblasts were derived from bone (calvaria) of neonatal Sprague Dawley rats purchased from Charles River (Horsham, PA). All animals were handled at the AAALAC approved animal facility in The Commonwealth Medical College (Scranton, PA), according to the principles in the NIH Guide for the Care and Use of Laboratory Animals (U.S. Department of Health and Human Services, Publ. No. 86–23, 1985) and guidelines established by the IACUC of The Commonwealth Medical College (Scranton, PA). Adult animals were euthanized by CO_2_ inhalation followed by cervical dislocation. Neonate rats were euthanized by decapitation.

### Primary Osteoblast Cell Culture

Primary osteoblast cultures were obtained using neonatal rats as previously described [4, 10, 11]. Primary cells were isolated from parietal calvaria from which the periosteum and cranial sutures were removed to reduce non-osteoblast cell contamination. Calvaria pieces were subject to five sequential digestions of 5, 15, 15, 25, and 25 min at 37° C in a shaking water bath with 0.1% collagenase-P (Roche, San Francisco, CA)/ 0.25% trypsin (Mediatec, Thermo Fisher). The first two digestions are performed to remove non-osteoblast cells and are discarded. Osteoblast-enriched cell populations were obtained from the 3^rd^–5^th^ digestions of the calvarial pieces. These cells were plated in 100mm dishes (Falcon) at 5 × 10^5^ cells/plate in osteogenic media consisting of Earle’s Minimal Essential Medium (EMEM; Mediatec) supplemented with 10% fetal calf serum (FCS; Mediatec), 50μg/ml ascorbic acid (Sigma) and 10mM βcglycerophosphate (Sigma). The cells were incubated at 37°C with 5% CO_2_ with a change of media every three days until they reached 80% confluence. Cells were sub-cultured under identical conditions for utilization in experiments following the third passage. We have previously shown that these culture conditions result in an enhanced (>90%) population of cells committed to the osteoblast lineage using specific markers of osteoblast differentiation (e.g. Runx2, Osterix) [4, 11].

### Protein Isolation, Western Blotting and transfection

Lysate preparation, Immunoprecipitation, Western Blotting and trasfection were preformed as described [76]. Blots were incubated with primary and secondary antibodies following the manufacturers instructions. one of the following primary antibodies: AFAP1 (1:1000), phospho-SRC (1:1000), SRC (1:1000), Collagen I (1:1000), Collagen XII (1:1000), actin (1:5000), and CTGF (1:200), and then with the corresponding HRP-conjugated secondary antibody (1:20,000).

### Luciferase Assays

Luciferase activity was determined using the Dual-Glo luciferase assay (Promega, Madison, WI) according to the manufacturer’s instructions as described [11]. Briefly, primary osteoblasts were plated in a 96-well microplate (2.4 × 10^4^ cells/well), transfected with CCN2 promoter reporter vector and co-transfected with Renilla (internal control) luciferase vector. Following transfection, the cells were serum starved overnight and treated with TGF-nd co-transfected with Renilla (internal Luciferase activity was measured using a SpectraMax M5 Microplate Reader (Molecular Devices, Sunnyvale, CA). Relative luciferase activity was expressed as a ratio of firefly/renilla luminescence values. All samples were normalized to an untreated (cells only) or mock treated (empty vector or diluent only) control reaction.

### Data Analysis

For all quantitative data, analysis of variance (ANOVA) was employed to evaluate the effect of one variable on two or more independent groups. In the event of a significant group effect, individual pairs of means were compared using the Bonferroni post-hoc test. Data were calculated as mean + SEM, and in some cases, converted to percent of control. A value of p<0.05 was used to determine whether differences were statistically significant.
